# The Effect of Endophytic *Talaromyces pinophilus* on Growth, Absorption and Accumulation of Heavy Metals of *Triticum aestivum* Grown on Sandy Soil Amended by Sewage Sludge

**DOI:** 10.3390/plants10122659

**Published:** 2021-12-03

**Authors:** Amany A. El-Shahir, Noha A. El-Tayeh, Omar M. Ali, Arafat Abdel Hamed Abdel Latef, Naglaa Loutfy

**Affiliations:** 1 Department of Botany and Microbiology, Faculty of Science, South Valley University, Qena 83523, Egypt; nohaeltayeh@gmail.com (N.A.E.-T.); naglaaloutfy@yahoo.com (N.L.); 2 Department of Chemistry, Turabah University College, Turabah Branch, Taif University, P.O. Box 11099, Taif 21944, Saudi Arabia; om.ali@tu.edu.sa

**Keywords:** endophytic fungi, heavy metal, phytoremediation, sewage sludge, wheat

## Abstract

Sewage sludge improves agricultural soil and plant growth, but there are risks associated with its use, including high heavy metal content. In this study, experiments were carried out to investigate the role of endophytic *Talaromyces pinophilus* MW695526 on the growth of *Triticum aestivum* cultivated in soil amended with sewage sludge and its phytoremediation ability. *T. pinophilus* could produce gibberellic acid (GA) and stimulate *T. aestivum* to accumulate GA. The results showed that inoculation with *T. pinophilus* boosted plant growth criteria, photosynthetic pigments, osmolytes (soluble proteins, soluble sugars and total amino acids), enzymatic antioxidants (catalase, superoxide dismutase and peroxidase), K, Ca and Mg. On the other hand, it reduced Na, Na/K ratio, Cd, Ni, Cu and Zn in the growth media as well as in the shoot and root of *T. aestivum*. The results suggest that endophytic *T. pinophilus* can work as a barrier to reduce the absorption of heavy metals in *T. aestivum* cultivated in soil amended with sewage sludge.

## 1. Introduction

Plants and endophytes have a natural symbiotic relationship. Microbes that live inside a plant are called endophytic microorganisms. They get into a plant’s seeds, leaves, stems, and roots, and are not damaging to their hosts. According to reports, 90% of plants achieve symbiosis with microbes, including endophytic fungi [1].

The plant–endophyte relationship has significant benefits for the plant, including the following: (i) Increasing plant growth; (ii) Providing secondary metabolites for the plant; (iii) Increasing plant resistance to pests and diseases [2]. Several studies have shown the role of endophytic microorganisms as biofertilizers and biocontrol agents.

Microorganisms are essential for sustaining soil fertility and plant health. They can operate as biofertilizers and boost biotic and abiotic stress resistance [3]. The direct benefits of interacting with endophytic fungi include increased nutrient acquisition and phytohormone levels in the plant, all of which are directly associated with increased biomass production, root system development, plant height, weight reproduction, and yield. Endophytes are known as biofertilizers because of these properties [4,5].

Sludge from sewage treatment is a biological byproduct that boosts macronutrients and organic matter in agricultural and degraded soils, and it is commonly utilized for plant fertilization since it allows organic matter to be recycled back into the soil. Concerns about the use of municipal sewage sludge for plant fertilization have grown in response to elevated metal (loid) concentrations in some sewage-amended soils, which could have negative consequences for the ecosystem and human health. Data on the cleaning strategies of sewage-affected soils are required due to the possible harmful repercussions of metal presence [6]. Heavy metals are dangerous to human health because of their carcinogenicity, mutagenicity and cytotoxicity [7]. It has been found that, in comparison to the high costs of chemical and physical remediation procedures, which can be harmful to ecosystems, phytoremediation provides an environmentally friendly and low-cost mechanism for purifying contaminated soils [8]. Phytoremediation preserves the biological action and physical structure of soils, allowing for metal separation [9].

Phytoremediation is the process of reducing pollutant concentrations and/or their toxic effects on the environment by using plants and associated microbes in soil [10]. The remediation of heavy-metal-contaminated areas is gaining traction, but it is a difficult task due to the inability of metals to dissolve, and their risks are worsened by their long-term presence in the environment [11]. Phytoremediation may be used to remove and/or stabilize inorganic and organic contaminants, and it has been hailed as a viable alternative to traditional remediation procedures because it causes less site disruption, costs less and has greater public acceptance. However, a number of challenges remain in the field-scale application of phytoremediation, such as slow growth and tiny biomass, phytotoxicity, evapotranspiration of volatile pollutants and so on [12]. Microbe-assisted phytoremediation can be used to address these issues [13,14]. Endophytic fungi are common, and some have been proven to boost phytoremediation efficacy [15,16,17,18]. Many endophytic fungi belonging to *Peyronellaea* sp. were discovered in previous studies. They were isolated from heavy-metal-contaminated as well as non-contaminated areas, and some of them demonstrated high Pb and Zn tolerance [15]. The heavy-metal-tolerant fungal isolates could also boost phytoremediation effectiveness and show significant intraspecific variabilities. In polluted copper and cadmium, the fungal endophyte *Penicillium funiculosum* LHL06 grew faster than other endophytes. In comparison to cadmium, *P. funiculosum* demonstrated a high biosorption capacity for Cu. Inoculation of the host *Glycine max* L. plants with *P. funiculosum* during Cu stress (100 µM) was used to test the endophyte–metal–plant interaction [19]. *Talaromyces pinophilus* was found to be a common phyllosphere, phylloplane and endophyte in different medicinal plants in earlier studies [20,21,22]. Due to the presence of bioactive substances, *T. pinophilus* has commonly been utilized as an endophyte [23,24]. In this study, we aimed to use *Talaromyces pinophilus* as an endophyte to perform phytoremediation in the presence of sewage sludge during wheat (*Triticum aestivum*) cultivation.

## 2. Materials and Methods

### 2.1. Fungal Isolation and Identification

*Rosmarinus officinalis* leaves were collected from different sites in Upper Egypt. The samples were properly cleaned using tap water before being rinsed with sterile distilled water. Surface sterilisation was achieved by immersing samples in 75% ethanol for 1 min, 4% sodium hypochlorite for 3 min and then 75% ethanol for 30 s. After that, the leaves were rinsed in sterile distilled water and dried in the shade. Leaves were cut into small pieces and placed on previously poured glucose-agar Czapek’s plates, which were then cultured in a microbiological incubator at 28 °C for 7 days until the desired fungi grew [25].

### 2.2. Morphological Identification of the Fungal Isolate

On czapek yeast extract agar (CYA), the fungus was inoculated in the center of 9 cm Petri dishes and incubated for seven days at 28 °C. The isolate was identified using a light microscope to examine colony morphology, and microscopic examination of hyphae, conidiophores, and conidia was carried out using LABOMED CxL (Labo America, Inc. Fermont, CA, USA). The photos were obtained with a camera that was attached to the (Leica Icc 50). The ImageJ program was used to measure the conidiophore, metulae, phialide and conidia. Fungi were first recognized using keys designed by [26,27] based on culture and microscopic characteristics. 

### 2.3. Molecular Identification

The molecular identification of fungi was conducted using the polymerase chain reaction (PCR) amplification of the ribosomal internal transcribed spacer (ITS) region, as described previously by [28]. The DNA amplification and sequencing were carried out according to the protocol reported by [29]. The obtained sequence of the desired fungus was deposited in the nucleotide sequence database of GenBank to analyze it using BLAST search program at the NCBI website: http://blast.ncbi.nlm.nih.gov/Blast.cgi, 21 November 2021. Sequences were aligned by MUSCLE and introduced to MEGA-X version 10.2.2 software for phylogenetic analysis. The evolutionary history was inferred using the Neighbor-Joining method [30]. The optimal tree is shown. The evolutionary distances were computed using the Maximum Composite Likelihood method [31]. Evolutionary analyses were conducted in MEGA X [32].

The QIA quick PCR Product extraction kit was used to purify the PCR products (Valencia, Spain, Qiagen). The sequence reaction was performed with a Perkin-Elmer Bigdye Terminator V3.1 cycle sequencing kit, and the product was purified with a Centrisep spin column. The PCR yields were sequenced by Elim Biopharmaceuticals Inc. (Biotechnology company in Hayward, CA, USA) and sequence identity to Gen Bank accessions was established using a BLAST analysis (Basic Local Alignment Search Tool) [33].

### 2.4. Fungal Inoculum Preparation

After 3 h of soaking in distilled water, 100 g of wheat seeds were placed in 250 mL Erlenmeyer flasks and autoclave sterilized. The flasks were then inoculated with the endophytic fungus and cultured at 28 °C for 10 days until the appropriate fungi appeared.

### 2.5. Extraction and Determination of Phytohormones

Seven-day-old mycelium fungal strain was inoculated in a separate Erlenmeyer flask containing 250 mL of autoclaved Potato Dextrose Broth. Flasks were incubated at 25 ± 1 °C for 7 days. After 7 days, the culture filtrate of each flask was filtered through Whatman no. 42 filter paper. The pH of the filtrate was adjusted to 2.5–3.0 by adding 0.1 N HCl.

Extraction and determination of phytohormones on desired endophytic fungi and in seedlings for the eight treatments of *T. aestivum* were conducted by the method explained earlier by [34].

### 2.6. Plant Material and Growth Conditions

Sewage sludge was gathered from Qena governorate’s Wastewater Treatment Plants (WTP) (El-Salhya). Dry sewage sludge was brought straight to the lab and manually ground to pass through a 2 mm sieve before being used in the studies. Sand soil was obtained from the Qena desert area at the same time. The pot experiments were arranged in a randomized complete block design with three replicates for each treatment. The growing medium was made by combining sandy soil with various quantities of sewage sludge from the Qena sewage wastewater treatment plant (2609 19.8 N–3246 35.8 E) and an endophytic fungus. The soil had a sandy loam texture consisting of 72% coarse sand, 8% silt and 18% clay. Treatments were designated as shown in Table 1.

All of the experiments took place in a sunlight greenhouse with natural light, a 8/16 h day/night cycle at 25/17 °C, and 30–50% relative humidity, and the field capacity was determined for each sludge level before cultivating the seeds.

The plant material utilized was wheat (*Triticum aestivum* cv. Bani Sweif 5). The Agronomy Department of South Valley University’s Faculty of Agriculture provided the seeds. Ten wheat seeds of uniform size were placed in each pot and irrigated with distilled water regularly until the field capacity was reached. Each sludge level’s field capacity was determined, and the amount of water lost per day was compensated. The plants were given ten weeks to grow. During germination and growth, all plants were given 500 mL of Hoagland’s solution (0.5 strength) [35]. Hoagland’s solution was added partially at 10-day intervals (approximately 50 mL per interval).

Water extracts of a 1:5 (soil:water) ratio of all sludge levels were prepared according to the Staff of US Salinity Lab [36]. Similar extracts were also prepared for sludges and sand. The pH values for water extracts of soils with different sludge levels were measured using a pH meter. The electrical conductivity (Ec) in water extracts of sludge samples was measured using a conductance meter.

The dichromate oxidation technique, according to [37], was used to evaluate organic content in the sludge and the soil + sludge samples.

### 2.7. Determination of Growth Parameters

The roots and shoots of the collected plant seedlings were separated. For each sample, water content, shoot length and root length were measured. The parts of the harvested plant were instantly frozen and kept at −30 °C for biochemical analysis. To determine the constant weight for dry mass estimation, some parts of the samples were rapidly dried in an oven at 70 °C.

### 2.8. Determination of Photosynthetic Pigments

According to [38], photosynthetic pigments (chlorophyll a, b, and carotenoids) were extracted from fresh leaves and determined.

### 2.9. Determination of Organic Solutes

According to the method of [39], soluble proteins content was determined. Total amino acids were extracted from plant tissues and determined [40]. Soluble sugars content was estimated according to [41] by the anthrone sulphuric acid method.

### 2.10. Antioxidant Enzymes Activity

0.5 g of plant tissue was homogenized in 1 mL K-phosphate buffer pH 7, 0.1 mM Na2EDTA and 1% PVP, and then centrifuged at 10,000 rpm for 20 min at 4 °C. The enzyme assay was performed on the supernatant. The activity of catalase (CAT) was measured using the method described in [42]. The activity of peroxidase (POD) was measured according to [43]. The activity of superoxide dismutase (SOD) was measured using the method described in [44].

### 2.11. Determination of Mineral Ions and Heavy Metals

The plants’ shoots and roots were weighed, oven-dried and then powdered for extraction and chemical analysis at the end of the experiment. Mineral analysis was performed on shoot and root extracts for Na, K, Ca and Mg, according to [36]. The mixed acid digestion process reported by [45] was utilized to prepare extracts of various sewage sludge levels or plant components for heavy metals analysis. Using an atomic absorption spectrophotometer, the Cu, Zn, Cd and Ni content was calculated. The flame emission technique [46] was used to determine the Na and K content. The volumetric determination of Ca and Mg was performed using the versene titration method, as described by [47].

### 2.12. Determination of the Bioconcentration Factor (BCF) and Translocation Factor (TF)

The bioconcentration factor (BCF), an index of metals extracted from soil and accumulated in plant tissues, is the ratio of plant to soil metal concentration. The translocation factor (TF), an index of metals translocated from the root to the shoot, is the ratio of plant shoot to root metal concentration [48]. A TF > 1 means that the plant has a greater capacity to transport metals from the root to above-ground biomass:

BCF = C_plant_/C_soil_

TF = C_shoot_/C_root_

where C_plant_ is the metal concentration in the plant (µg g^−1^), C_soil_ is the metal concentration in soil, C_shoot_ is the metal concentration in the plant shoot (µg g^−1^), and C_root_ is the metal concentration in plant root.

### 2.13. Statistical Analysis

Statistics were carried out using SPSS release 23 (SPSS Inc., Chicago, IL, USA). Analysis of variance (ANOVA) was carried using a general one-way model, and Duncan’s test was used for comparison between means. Differences in plants and growth media between treatments were tested by one-way analysis of variance (ANOVA), and Duncan’s test was used as a post-hoc test. The student *t*-test was used to compare growth media properties before planting and after harvesting.

## 3. Results

### 3.1. Isolation and Morphological Identification of the Fungal Isolate

**Figure 1:**
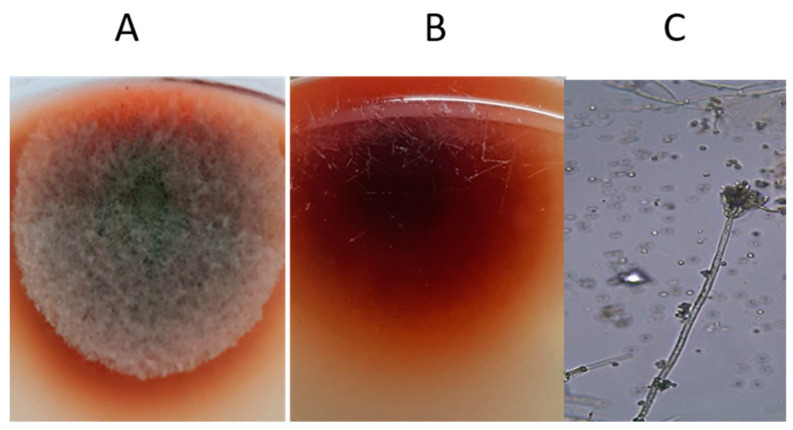
*Talaromyces pinophilus* MW695526 (**A**) Surface or observe view of 7-days old culture on CYA medium at 25 °C, (**B**) Bottom or reverse view and (**C**) Conidiophores and conidia.

The endophytic fungus *Talaromyces pinophilus* was isolated from *Rosmarinus officinalis* leaves. The fungus was first identified based on colony patterns, growth texture and micromorphological features. On czapek yeast extract agar (CYA), following a 7-day incubation period at 25 °C, the average colony diameter was 1.7 cm, with greyish green to dull green mycelia observed. The texture of the growth was loosely floccose and funiculus, particularly in the middle (Figure 1A). The reverse of the colonies was greyish orange (Figure 1B). Conidiophores (24.15 × 1.30 μm) were smooth and symmetrically biverticillate. They were smooth-walled and had three to six metulae (2.66 × 0.65 μm) that bore phialides (2.27 × 0.52 μm). Conidia were smooth, globose to subglobose and 0.62 μm in diameter (Figure 1C). The structures of ascomata were not present.

### 3.2. Nucleotide Sequence Accession Number and Phylogenetic Analysis

**Figure 2:**
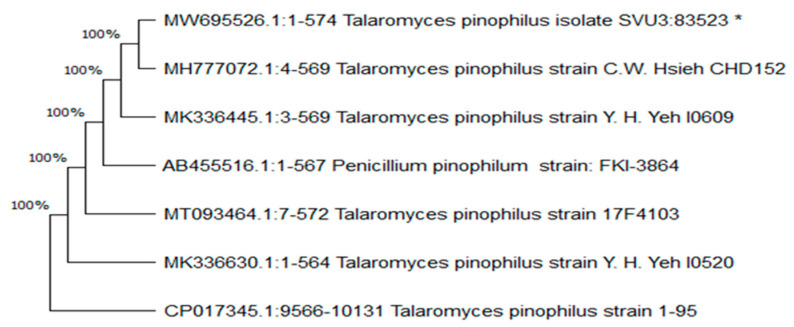
Neighbor-joining phylogenetic tree generated from *T. pinophilus* and its related isolates. * *T. pinophilus* used in this study.

*T. pinophilus* obtained sequence (internal transcribed spacer 1, 5.8S sequence) was deposited in the GenBank nucleotide sequence database under accession number MW695526. The BLAST survey confirmed the morphological identification. The closest match (99.65–99.82% similarity) in the NCBI GenBank database was detected among different *T. pinophilus* strains (Table 2). Moreover, the amplified and sequenced ITS region (574 base pair) of *T. pinophilus* MW695526 had a 99.82% identity to *T. pinophilus* strain MK336445. The molecular phylogenetic tree was constructed using NCBI-BLAST (Figure 2). The BLAST data confirmed the variation of *Talaromyces* strains.

### 3.3. Phytohormones

**Figure 3:**
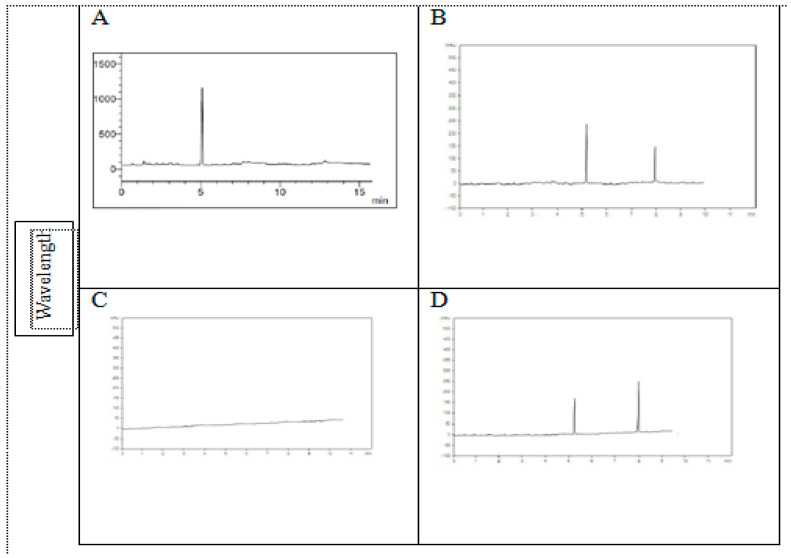
HPLC chromatograms obtained for (**A**) gibberellic acid content of *Talaromyce pinophilus*, (**B**) *Triticum aestivum* plants grown in sandy soil as control, (**C**) plants grown in a mixture of sandy soil 25% + sewage sludge 75% and (**D**) plants grown in a mixture of sandy soil 25% + sewage sludge 75% + *T. pinophilus*.

*T. pinophilus* had the ability to produce gibberellic acid (GA) at a retention time of 5 with the concentration 5.77 µg mL^−1^. For *Triticum aestivum* plants grown in mixtures of sandy soil and sewage sludge mixed with *T. pinophilus*, T6 (75% sandy soil + 25% sewage sludge + *T. pinophilus*) had the highest value of GA (5.36 µg mL^−1^), followed by T8 (25% sandy soil + 75% sewage sludge + *T. pinophilus*), which recorded a value of 5.22 µg mL^−1^ versus the control. In T4 (25% sandy soil + 75% sewage sludge), no GA was detected. For abscisic acid, the highest production was at T8 (25% sandy soil + 75% sewage sludge + *T. pinophilus*), with a value of 7.13 µg mL^−1^ recorded, followed by T5 (100% sandy soil + *T. pinophilus*), 5.14 µg mL^−1^ (Figure 3). Abscisic acid was not detected in T4, T6, or T7 (Table 3).

### 3.4. Water Content (WC) and Growth of T. aestivum Plants Grown on Different Sewage Sludge Levels and Phytoremediation by T. pinophilus

**Figure 4:**
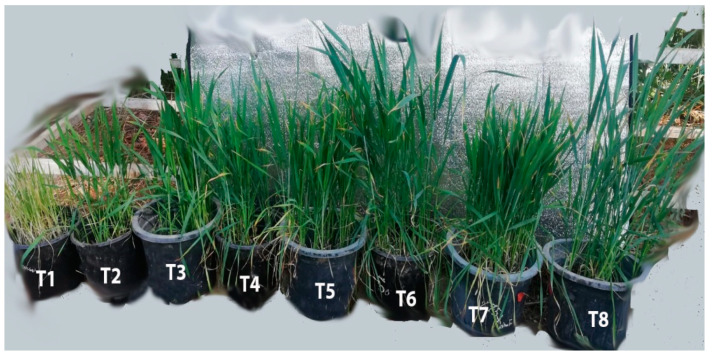
*Triticum aestivum* plants grown on mixtures of sandy soil and sewage sludge and the same treatment mixed with *T. pinophilus*. 100% sandy soil (T1), 75% sandy soil + 25% sewage sludge (T2), 50% sandy soil + 50% sewage sludge (T3), 25% sandy soil + 75% sewage sludge (T4), 100% sandy soil + *T. pinophilus* (T5), 75% sandy soil + 25% sewage sludge + *T. pinophilus* (T6), 50% sandy soil + 50% sewage sludge + *T. pinophilus* (T7), 25% sandy soil + 75% sewage sludge + *T. pinophilus* (T8).

Amendment of sandy soil with different sewage sludge levels caused a marked increase in all of these parameters of the *T. aestivum* plants as compared with control ones (Figure 4).

**Figure 5:**
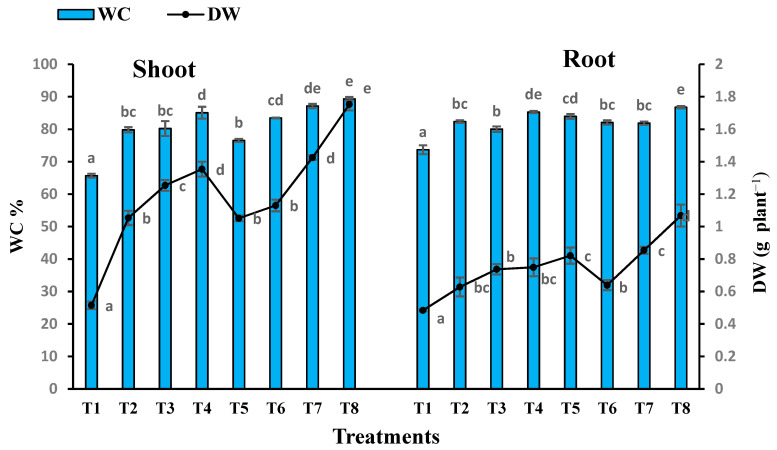
Water content (WC) and dry weight (DW) of shoot and root of *T. aestivum* plants grown on mixtures of sandy soil and sewage sludge and the same treatment mixed with *T. pinophilus*. 100% sandy soil (T1), 75% sandy soil + 25% sewage sludge (T2), 50% sandy soil + 50% sewage sludge (T3), 25% sandy soil + 75% sewage sludge (T4), 100% sandy soil + *T. pinophilus* (T5), 75% sandy soil + 25% sewage sludge + *T. pinophilus* (T6), 50% sandy soil + 50% sewage sludge + *T. pinophilus* (T7), 25% sandy soil + 75% sewage sludge + *T. pinophilus* (T8). Data are means ± SE, n = 3. Different letters indicate significant differences between treatments at *p* < 0.05 according to Duncan test.

**Figure 6:**
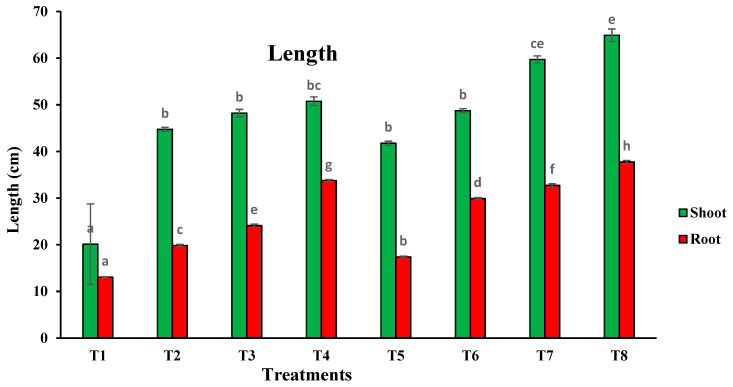
Length of shoot and root of *T. aestivum* plants grown on mixtures of sandy soil and sewage sludge and the same treatment mixed with with *T. pinophilus*. 100% sandy soil (T1), 75% sandy soil + 25% sewage sludge (T2), 50% sandy soil + 50% sewage sludge (T3), 25% sandy soil + 75% sewage sludge (T4), 100% sandy soil + *T. pinophilus* (T5), 75% sandy soil + 25% sewage sludge + *T. pinophilus* (T6), 50% sandy soil + 50% sewage sludge + *T. pinophilus* (T7), 25% sandy soil + 75% sewage sludge + *T. pinophilus* (T8). Data are means ± SE, n = 3. Different letters indicate significant differences between treatments at *p* < 0.05 according to Duncan test.

Several growth parameters of *T. aestivum* were determined, including root and shoot dry weight, root and shoot length and water content (Figure 5 and Figure 6). Inoculation with *T. pinophilus* enhanced the water content in the shoot of *T. aestivum* by about 16.55% (T5), 4.52% (T6), 8.63% (T7) and 5.02% (T8), respectively, as compared with the same level of sewage sludge without the fungus. Moreover, there was an increase in the WC in the root of the plants in the soil levels colonized by *T. pinophilus.* On the other hand, inoculation with *T. pinophilus* enhanced the length of the shoot of *T. aestivum* by 108% (T5), 9% (T6), 24% (T7) and 28% (T8), respectively, versus the same level of sewage sludge without the fungus, and enhanced the length of the root by 33% (T5), 51% (T6), 36% (T7) and 11% (T8), respectively. The dry weight also increased by increasing the sewage sludge levels as compared with the control ones. Colonization of the soil levels with *T. pinophilus* enhanced the dry weight of the shoot of *T. aestivum* by 104.08% (T5), 7.31% (T6), 5.24% (T7) and 29.46% (T8), respectively, against the same level of sewage sludge without the fungus, and by 69.63% (T5), 1.75% (T6), 15.88% (T7) and 42.59% (T8) in the case of the root.

### 3.5. Photosynthetic Pigments of T. aestivum Plants Grown on Different Sewage Sludge Levels and Phytoremediation by T. pinophilus

**Figure 7:**
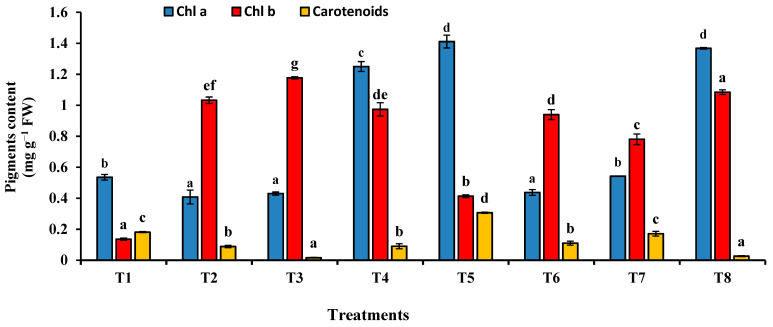
Chlorophyll (Chl) a, Chl b and carotenoids content in fresh leaves of *Triticum aestivum* plants grown on mixtures of sandy soil and sewage sludge and the same treatment mixed with *T. pinophilus*. 100% sandy soil (T1), 75% sandy soil + 25% sewage sludge (T2), 50% sandy soil + 50% sewage sludge (T3), 25% sandy soil + 75% sewage sludge (T4), 100% sandy soil + *T. pinophilus* (T5), 75% sandy soil + 25% sewage sludge + *T. pinophilus* (T6), 50% sandy soil + 50% sewage sludge + *T. pinophilus* (T7), 25% sandy soil + 75% sewage sludge + *T. pinophilus* (T8). Data are means ± SE, n = 3. Different letters indicate significant differences between treatments at *p* < 0.05 according to Duncan test. FW, fresh weight.

The effect of the sewage sludge on the photosynthetic pigments is illustrated in Figure 7. Sewage sludge amendment at all levels induced a noticeable decrease in Chl a and carotenoids, while there was a significant increase in Chl b. The drastic reduction in the content of Chl a and carotenoids was observed at the level 50% sewage sludge. On the other hand, there was an observable increase in Chl a and carotenoids after incubation of the soil with *T. pinophilus,* as compared with the same level of sewage sludge without the fungus. The enhancement effect of *T. pinophilus* on Chl b was observed only at the highest level of sewage sludge (75%).

### 3.6. Osmolytes of T. aestivum Plants Grown on Different Sludge Levels and Phytoremediation by T. pinophilus

**Figure 8:**
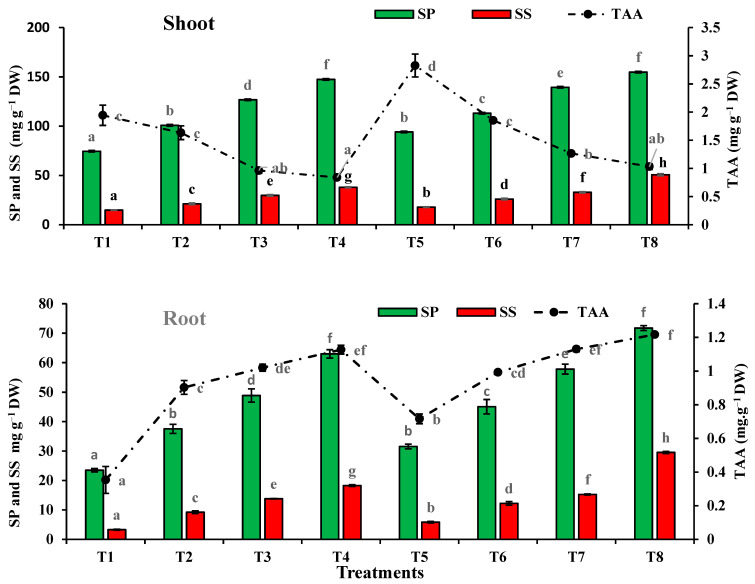
Soluble proteins (SP), soluble sugars (SS) and total amino acids (TAA) content in shoot and root of *Triticum aestivum* plants grown on mixtures of sandy soil and sewage sludge and the same treatment mixed with *T. pinophilus*. 100% sandy soil (T1), 75% sandy soil + 25% sewage sludge (T2), 50% sandy soil + 50% sewage sludge (T3), 25% sandy soil + 75% sewage sludge (T4), 100% sandy soil + *T. pinophilus* (T5), 75% sandy soil + 25% sewage sludge + *T. pinophilus* (T6), 50% sandy soil + 50% sewage sludge + *T. pinophilus* (T7), 25% sandy soil + 75% sewage sludge + *T. pinophilus* (T8). Data are means ± SE, n = 3. Different letters indicate significant differences between treatments at *p* < 0.05 according to Duncan test. DW, dry weight.

The data presented in Figure 8 showed that amendment of the sandy soil with different sewage sludge levels caused a significant increase in soluble proteins (SP) and soluble sugars (SS) in the shoot and root of *T. aestivum* plants in comparison with the control plants. On the other hand, total amino acids (TAA) in the shoot were only significantly reduced (by 57%) due to the application of the high level of sewage sludge. The osmolyte content levels were higher in the shoot than in the root. The increase in the SP in the wheat shoot and root at the higher level of sewage sludge (75%) was 1.98-fold and 2.68-fold, respectively, versus the control, and was 2.56-fold and 5.56-fold in the case of the SS in the wheat shoot and root, respectively, as compared with the control. Meanwhile, the increase in the TAA content in the wheat root was 3.19-fold, as compared with the control.

Further increases in the SP, SS and TAA in the shoot and root of *T. aestivum* plants were registered in plants incubated with *T. pinophilus*, under all levels of sewage sludge amendment. Under the influence of the highest level of sewage sludge (75%), increases in SP, SS and TAA were 3.05-fold, 8.98-fold and 3.49-fold in roots and 2.08-fold, 3.41-fold and 0.53-fold in shoots, respectively, as compared with the control.

### 3.7. Antioxidant Enzymes of T. aestivum Plants Grown on Different Sludge Levels and Phytoremediation by T. pinophilus

**Figure 9:**
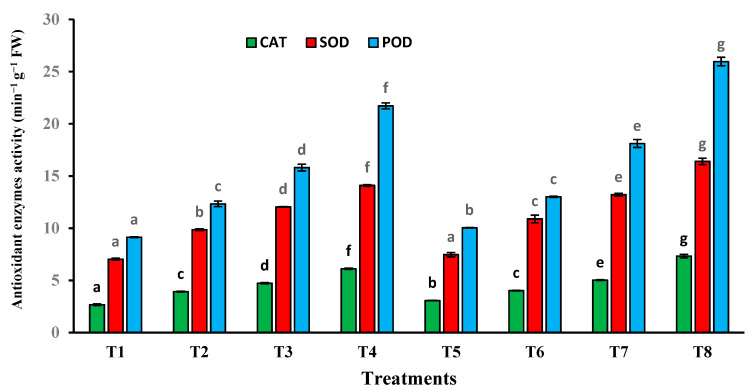
Catalase (CAT), superoxide dismutase (SOD) and peroxidase (POD) activity in fresh leaves of *T. aestivum* plants grown on mixtures of sandy soil and sewage sludge and the same treatment mixed with *T. pinophilus*. 100% sandy soil (T1), 75% sandy soil + 25% sewage sludge (T2), 50% sandy soil + 50% sewage sludge (T3), 25% sandy soil + 75% sewage sludge (T4), 100% sandy soil + *T. pinophilus* (T5), 75% sandy soil + 25% sewage sludge + *T. pinophilus* (T6), 50% sandy soil + 50% sewage sludge + *T. pinophilus* (T7), 25% sandy soil + 75% sewage sludge + *T. pinophilus* (T8). Data are means ± SE, n = 3. Different letters indicate significant differences between treatments at *p* < 0.05 according to Duncan test. FW, fresh weight.

Generally, the activities catalase (CAT), superoxide dismutase (SOD) and peroxidase (POD) increased under the effect of different sewage sludge levels and the inoculation of plants with *T. pinophilus* (Figure 9). In comparison with the control plants, under the influence of a 75% sewage sludge level, a marked increase in CAT activity (2.29-fold), SOD activity (2.00-fold) and POD activity (2.38-fold) was recorded. In addition, a further increase in the activities of CAT, SOD and POD was registered in plants incubated with *T. pinophilus*, under the higher level of sewage sludge amendment. Under the effect of this treatment, the activities of CAT, SOD and POD increased by 2.75-fold, 2.33-fold and 2.84-fold, respectively.

### 3.8. Translocation and Content of Minerals in Wheat Plant Cations

**Figure 10:**
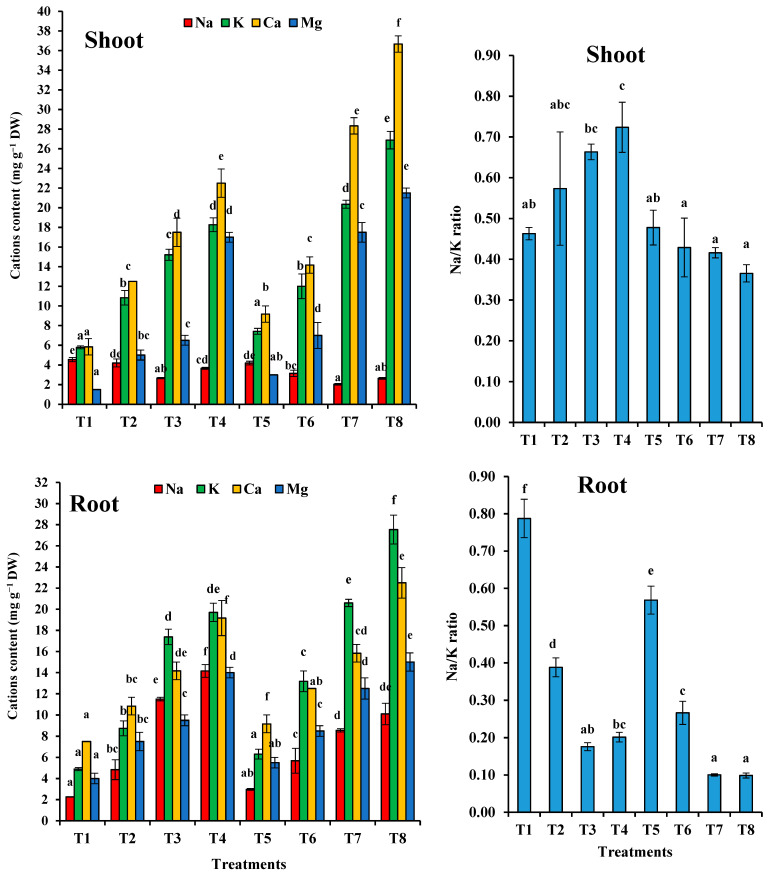
Cations content and Na/K ratio in shoot and root of *Triticum aestivum* plants grown on mixtures of sandy soil and sewage sludge and the same treatment mixed with *T. pinophilus*. 100% sandy soil (T1), 75% sandy soil + 25% sewage sludge (T2), 50% sandy soil + 50% sewage sludge (T3), 25% sandy soil + 75% sewage sludge (T4), 100% sandy soil + *T. pinophilus* (T5), 75% sandy soil + 25% sewage sludge + *T. pinophilus* (T6), 50% sandy soil + 50% sewage sludge + *T. pinophilus* (T7), 25% sandy soil + 75% sewage sludge + *T. pinophilus* (T8). Data are means ± SE, n = 3. Different letters indicate significant differences between treatments at *p* < 0.05 according to Duncan test. DW, dry weight.

The changes in major cation contents (Na, K, Ca, and Mg) in various organs of the wheat plants grown in soil amended with different levels of sewage sludge and phytoremediation by *T. pinophilus* are represented in Figure 10. Na content was accumulated significantly in the root. The amount of accumulation was shown to be proportional with the sewage sludge levels, but inoculation of with *T. pinophilus* reduced the Na content in the root and shoot of the studied plants. In the case of shoots, the content was decreased by increasing the sewage sludge level (the lowest accumulation was at a 50% sewage sludge level) and incubating the soil sewage sludge levels with *T. pinophilus* significantly reduced the Na content in the shoot. There were significant differences reported in the Na content of each organ at all levels.

The content of K was significantly increased in shoot and root at T2, T3 and T4 of sewage sludge levels by (1.87-fold, 2.63-fold) and (3.16-fold, 1.79-fold) and (3.55-fold and 4.03-fold), respectively, compared to the control plants (Figure 10). Inoculation with *T. pinophilus* caused a significant accumulation of K content in plants treated with the same level of sewage sludge, as compared with the control plants T6 (2.08-fold), T7 (3.52-fold), and T8 (4.65-fold) in the case of shoot, and T6 (2.70-fold), T7 (4.21-fold) and T8 (5.63-fold) in the case of root.

The data presented in Figure 10 shows that the Na/K ratio progressively increased in root as the sewage sludge level increased. Colonization with *T. pinophilus* was accompanied by a significant decrease in the Na/K ratio in the root and shoot of *T. aestivum* plants. 

Generally, the Ca and Mg contents in the control plants were lower than the plants grown in different sewage sludge levels for both organs (Figure 10). Ca and Mg accumulated in the shoot more than in the root of the studied plants. Inoculation with *T. pinophilus* increased the Ca and Mg content in the root and shoot. The highest increase was recorded at T8 in the root and shoot of the studied plants.

### 3.9. Effects of Sewage Sludge and T. pinophilus on Growth Media

Increasing the sewage sludge proportion acidified the growth media before planting (ANOVA, F = 177.84, *p* < 0.001) and after harvesting (ANOVA, F = 521.54, *p* < 0.001). The pH was always higher before planting than after harvesting (*t*-test, *p* < 0.05), except for T7 (*t*-test = −0.06, *p* = 0.96) and T8 (*t*-test = −1.31, *p* = 0.26) (Table 4). Increasing the sewage sludge content in the growth medium stimulated increases in soil organic matter before planting (ANOVA, F = 177.84, *p* < 0.001) and after harvesting (ANOVA, F = 521.54, *p* < 0.001). Organic matter was higher before planting than after harvesting (*t*-test, *p* < 0.001) (Table 4).

The contents of Cu, Zn, Cd and Ni increased with increasing sewage sludge content before planting (ANOVA, *p* < 0.001), but inoculation with *T. pinophilus* after harvesting resulted in a decrease in the contents of Cu, Zn, Cd and Ni (ANOVA, *p* < 0.001) (Table 4). Generally, the contents of K, Ca and Mg increased gradually with increasing sewage sludge content before planting, and inoculation with *T. pinophilus* resulted in higher accumulations of the necessary elements (K, Ca and Mg) (ANOVA, *p* < 0.001), with these decreasing after harvesting (ANOVA, *p* < 0.001; *t*-test, *p* < 0.05) (Table 4). The content of Na increased with increasing sewage sludge content before planting (ANOVA, *p* < 0.01), while a decrease in the content of Na after inoculation with *T. pinophilus* was observed before and after harvesting (ANOVA, *p* < 0.001) (Table 4). The contents of Cu, Ni, Zn, Cd, Ca, Mg, and K were lower after harvesting than before planting for every treatment (*t*-test, *p* < 0.05) (Table 4). The contents of Cu, Ni, Zn and Cd were lower after harvesting than before planting, especially for the soil media incubated with the endophyte fungus for every treatment (*t*-test, *p* < 0.05) (Table 4).

**Figure 11:**
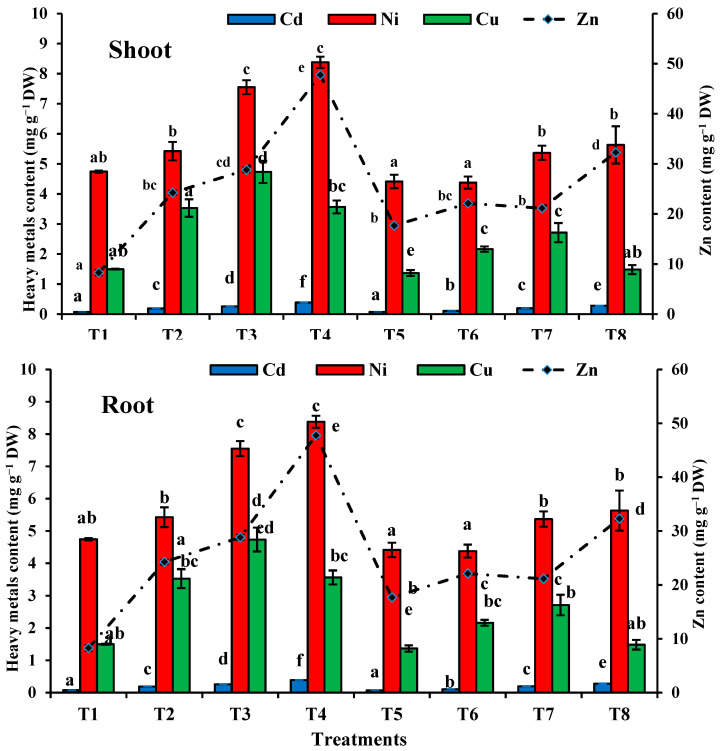
Heavy metals content in shoot and root of *T. aestivumplants* grown on mixtures of sandy soil and sewage sludge and the same treatment mixed with *T. pinophilus*. 100% sandy soil (T1), 75% sandy soil + 25% sewage sludge (T2), 50% sandy soil + 50% sewage sludge (T3), 25% sandy soil + 75% sewage sludge (T4), 100% sandy soil + *T. pinophilus* (T5), 75% sandy soil + 25% sewage sludge + *T. pinophilus* (T6), 50% sandy soil + 50% sewage sludge + *T. pinophilus* (T7), 25% sandy soil + 75% sewage sludge + *T. pinophilus* (T8). Data are means ± SE, n = 3. Different letters indicate significant differences between treatments at *p* < 0.05 according to Duncan test. DW, dry weight.

The total uptake (U) of every element by *T. aestivum* increased greatly when the sewage sludge content in the growth medium was increased. The maximum U values were recorded for T4, where the highest accumulated metal was Zn, followed by Ni > Cu > Cd. The metal accumulation under T4 was more than 5.77 times that for Zn, and 1.77 times that for Ni, and the accumulation of Cd under T6 was more than 5.23 times and 2.38 times that for Cu when compared with T1. Inoculation with *T. pinophilus* decreased the accumulation of most of the heavy metals in T5, T6, T7 and T8, except in the case of Zn in T5 (Figure 11).

The TF was lower than 1 for Zn, Cd, Ni and Cu in all treatments, except in T3 in the case of Cu, and in T1 and T5 in the case of Ni, which showed TF values of more than 1 (Table 5). The highest TF values were shown at T5 for Cu (1.081). On the other hand, the BCF increased with the addition of sewage sludge. Incubation of the soil levels with *T. pinophilus* increased the BCF. The highest BCF values were shown at T6 for Zn (2.613). The BCF of the metals showed gradual increases with increasing sewage sludge content (Table 5).

### 3.10. The Content and Distribution of Heavy Metals in T. aestivum

The data presented in Figure 11 show that in general, the sewage sludge level treatments increased Cd, Ni, Cu and Zn accumulation in *T. aestivum* plants compared to the control. 

Figure 11 shows that the modulation of the sandy soil with the sewage sludge increased gradually the content of Cd in the roots and shoots of the wheat plants. Colonization of *T. pinophilus* with the same levels of the soil caused a reduction in the content of Cd (1.35%, 42.55%, 25.51% and 27.65%, and 17.07%, 11.89, 39.02% and 51.83% in T5, T6, T7 and T8 in the shoot and root of the plants, respectively).

There was a significant increase in the Ni content in both the shoot and root of the wheat plants after the sewage sludge levels were increased. Colonization with *T. pinophilus* decreased the content of Ni significantly, as compared with the same levels of the soil sludge without colonization (T1 (4.74 mg g^−1^ DW), T2 (5.42 mg g^−1^ DW.), T3 (7.55 mg g^−1^ DW) and T4 (8.38 mg g^−1^ DW.) without fungus, respectively, and T1(4.41 mg g^−1^ DW), T2 (4.38 mg g^−1^ DW), T3 (5.37 mg g^−1^ DW) and T4 (5.63 mg g^−1^ DW) with fungus, respectively in the shoots. Meanwhile, in the roots of the studied plants, the fungus caused a reduction in the content of Ni, as compared with the same soil levels without the fungus.

Cu recorded its maximum increase in the shoots of the wheat (2.15-fold higher than the control at T3 (50% sewage sludge level). Meanwhile, in the roots of the wheat, the highest increase in Cu was 1.42-fold higher than the control at T4 (25% sandy soil + 75% sewage sludge). On the other hand, inoculation with the fungus and with the same levels significantly decreased the content of Cu as compared with the same levels without the fungus (Figure 11).

The maximum increase in Zn in the root and shoot of the wheat plants was 4.77-fold and 1.02-fold higher than the control, respectively, at a 75% sewage sludge level. Colonization of the soil levels with *T. pinophilus* caused decreases in the content of Zn in the shoots and roots of the studied plants, as compared with the same levels of sewage sludge without incubation with the fungus. 

## 4. Discussion

Our morphological identification of *T. pinophilus* is in agreement with [29] who revealed that after 7 days of incubation at 25 °C, the colonies on czapek yeast extract agar (CYA) measured 27–32 mm, were greyish-green to dull green and had white and yellow mycelia. The growth texture was floccose and funiculus. A grayish orange reverse was created by the colonies. The conidiophores were smooth and symmetrically biverticillate. The stipes (150–200 × 2–3 μm) were smoothly walled and had three to six metulae (10–11 × 2.5–3 μm) that bore acerose phialides (8.5–12 × 2–3 μm). Furthermore, smooth, globose to subglobose conidia with a diameter of 2–3μm were observed. It was confirmed through [49] that after 7 days of incubation in CYA, *T. pinophilus* colonies grew to a size of 16–31 mm. The colonies were floccose and funiculus, and had a deep green/blue-grey conidial area that was usually overgrown, with a white mycelium, yellow antimony and reverse apricot-orange colony observed. Many endophytes live in the roots of plants, and their cooperation may be crucial for plant growth, particularly in heavy-metal-contaminated environments. As a result, the role of endophytes in wheat plant tolerance to heavy metals must be exploited [50]. Many researchers have shown that *T. pinophilus* is a common endophyte found on medicinal plants and that it is a significant source of the enzymes, soluble pigments, and bioactive secondary metabolites needed for a variety of biotechnological applications [29,51,52].

Our phylogenetic study agrees with the findings of [53], who found that all *Talaromyces* strains (taken from cassava processing locations) in the database had a base sequence similarity of more than 98.8% to their new isolate LC128689. [54] The application of fungal phosphate solubilization, *T. pinophilus* treatment (M7), was found to be the most effective treatment for improving P availability and potato plant production. The results obtained by [55] suggested that using *Talaromyces flavus* may be able to promote cotton and potato growth characteristics. The authors of [56] reported that endophytes have been shown to improve wheat tolerance to abiotic stress and germination in endophyte-free second-generation seeds derived from stressed plants, which could be useful in agriculture.

Secondary metabolites are abundant in fungal endophytes. Plant hormones such as auxin, ethylene, GA, and indole acetic acid (IAA) are produced by many endophytes and are vital for plant growth regulation. Gibberellic acid is a highly effective phytohormone that controls plant development [57]. Our findings corroborated those of [58], who discovered that *T. pinophilus* isolate SCLB5 (KF913534) can synthesize a wide range of GAs. The isolate produced GA1 (0.465 ng mL^−1^), GA3 (1.808 ng mL^−1^), additional inactive GA9 (0.054 ng mL^−1^), and GA24 (0.044 ng mL^−1^) according to the GC/MS and HPLC results. It was established that it produced the same amounts of GA1, GA3, GA9 and GA24 as *G. fujikuroi*. It was discovered by [59] that *Penicillium funiculosum* LHL10 was resistant to heavy metals and produced more GA and IAA. Previous studies, in agreement with our results, have found that plant growth was stimulated in many plants by GA under a variety of abiotic stress conditions, such as drought, salinity, severe temperatures and heavy metal toxicity [60,61,62,63]. 

Sewage sludge has been used as a biofertilizer, and it has been found to have a positive effect on plant growth and soil biochemical and physical properties [64,65]. It also possesses plant growth regulators, auxins, enzymes, vitamins and hormones [66]. Several studies have found that adding sewage sludge to eroded soil can improve soil characteristics by changing the bulk density of the soil, aeration, and soil stabilization. Phytoremediation is the process of removing or rendering harmful environmental contaminants harm less by using green plants and related microbes in conjunction with proper soil amendments and agronomic approaches. In the present study, the effects of sewage sludge and the incubation of the soil with *T. pinophilus* were tested for their efficiency with regards to the growth of *Triticum aaestivum*, and the results clearly showed that sewage sludge treatments and soil incubation with *T. pinophilus* significantly improved the plant growth and related plant parameters. Our results showed a significant increase in water content, dry mass, photosynthetic pigments, some osmolytes and enzymatic antioxidants. These findings are consistent with those of [67]. This study found that many endophytic fungi might significantly boost the root-dry biomass of maize at higher Zn and Cd contents. According to the researchers, *P. citrinum* showed the ability to boost plant development, as well as to generate IAA, extracellular enzymes and auxin, all of which could help the inoculated plants grow faster. *Penicillium* sp. has been shown to boost plant growth by improving nutrient absorption [68]. Our findings are consistent with other studies that have shown that beneficial fungi improve plant growth even in disturbed and polluted environments [69,70,71].

Organic components encourage metabolic activity within the plant and improve the flow of metabolites from the roots to the leaves, potentially increasing the nutritious content of the plants [72]. In the present study, the sewage sludge significantly improved the mineral content and heavy metals in the wheat plants and affected the distribution of minerals and heavy metals in the roots and shoots. Our results showed that in general, there was a significant increase in K, Ca and Mg content in the wheat plants under sewage sludge treatments, as compared to the control. Inoculation with *T. pinophilus* led to an increase in mineral content. This increase in minerals may have been due to enhanced microbial activates, which increase nutrient availability and their uptake and increase root distribution, as described by [73,74]. This increase may also be attributable to the promotion of root growth by the sewage sludge and the soil’s colonization with *T. pinophilus,* which may have increased the soil’s capacity to absorb nutrients. Adding biofertilizers to organic fertilizers resulted in considerable increases in P and K uptake in the wheat plants, as mentioned in [75,76]. Our results showed a noticeable decrease in Na content in the shoots of *T. aestivum*. The higher Na levels observed in the roots as compared to the shoots in all treatments may have been due to the plant restricting the transport of Na to its upper shoots to minimize its detrimental effects [77]. Na and K were reversely distributed, and Na mostly accumulated in the roots, while more K was transported into the shoot of the plants. This may have been because K is needed for newly developed and photosynthetically active tissues [78]. The results of the present investigation indicated that the Na/K ratio was higher in the plant root than in the shoot. The Na/K ratio in the shoot of the plants was low because K is required by various enzymatic processes in the cytoplasm, so less Na competes with K for binding sites essential for cellular function, rather than substituting it [79]. Therefore, disruption of various enzymatic processes did not take place. Another reason could have been because high contents of K are needed for protein synthesis [80]. It is well established that potassium is generally the most prevalent ion in plant tissue and that it plays an important role in maintaining osmotic potential and activating certain enzymes, whereas the content of Na is very low and this ion plays no obvious metabolic role in most plants. In the present study, data indicated that the wheat plants retained Na in their roots and tended to transport more K into their shoot. However, when it comes to K uptake, most higher plants have evolved significant selectivity when compared to Na uptake.

The major factors limiting metal bioavailability are environmental conditions, particularly soil qualities [81]. The acceptable levels of heavy metals in soil have been determined as follows: Cr, Cu, and Pb (100 g g^−1^); Zn (300 g g^−1^); Fe (50,000 g g^−1^); Cd (3 g g^−1^); As (20 g g^−1^); Co and Ni (50 g g^−1^). As a result, the solubility of many metals increases in acidic soils [82], whereas high quantities of organic matter reduce their availability due to metal-organic complexity [83]. When we increased the sewage sludge levels in our study, organic matter increased, and the growth medium tended to reduce the negative effects of metal availability, raising the cation exchange capacity in the soil and making the nutrient accessibility for plant production more acidic, which may have resulted in organic matter decomposition [84]. The use of sewage sludge improved edaphic qualities, but it significantly raised metal contents in the soil, as previously documented in previous research [85,86]. The contents of Cd, Ni, Cu and Zn in the growing medium were lowered by colonizing the sewage sludge levels to levels below the World Health Organization’s (WHO) allowed limits for each treatment [81]. Metal transport to photosynthetic organs can be limited by root action [87]. The higher contents of metals (Cu, Zn, Cd, and Ni) in the root, rather than in the shoot, points to the high capacity of *T. pinophilus* to phytoextract such metals when growing under high contents. Heavy metal translocation rose progressively as sewage sludge content grew; however, these increments are thought to be artificial, because rising sludge contents made it easier for the plants to absorb such metals. The contents of heavy metals were reduced when the soil sludge levels were incubated with *T. pinophilus*. Our findings support *T. pinophilus’* ability to phytostabilize such metals when it is grown in high quantities.

The use of sewage sludge as an organic source resulted in a larger increase in the Zn, Cd, Cu and Ni content of the plants studied. Heavy metal accumulation was reduced in the plants colonized with the endophytic fungus. In all of the therapies studied, these effects were observed. The higher solubility of these metals in sewage-sludge-amended soil could be related to a decrease in soil pH [88,89]. Our findings are in agreement with those of [90], which found that the mobility of heavy metals can be increased by the addition of sewage sludge. The authors of [91] reported that the mobility of heavy metals increased with the addition of soluble organics. In the present study, the content of Cd in the *T. aestivum* plants grown in the soil amended with different levels of sewage sludge was higher than the allowable level of Cd at T3 and T4 in shoots and roots as set by the FAO/WHO-Codex Alimentarius commission, which is 0.2 mg kg^−1^ for leafy vegetables. Incubation with *T. pinophilus* reduced the content of Cd in the shoot and root of the studied plants on the border of the permissible level at T8 in shoot and root [92].

The Ni, Cu and Zn content of the *T. aestivum* plants in the shoot and root were lower than the maximum allowable level of Ni [77], and colonization of the different soil levels with *T. pinophilus* remediated the toxic metals in the shoots and roots of the studied plants.

The uptake of metals can be enhanced by increasing the mobility of metals with microorganisms that produce surfactants [93], siderophores [94] and organic acids [95], and the enhancement of the biomass of plants can be induced by using plant growth promoting rhizobacteria (PGPR) [96] and arbuscular mycorrhizal fungi (AMF) [97]. Bioaugmentation-assisted phytoextraction is a promising method for phytoremediation and has been reviewed by [98].

## 5. Conclusions

For phytoremediation, we can employ sewage sludge on the cultivation of *Triticum aestivum* in the presence of *Talaromyces pinophilus* MW695526. The ability to produce gibberellin was found in *T. pinophilus*. Gibberellic acid production is highest in *T. aestivum* plants grown in a sandy soil-sewage-sludge mixture with *T. pinophilus*. The results demonstrated that sewage sludge treatments and soil incubation with *T. pinophilus* boosted plant growth and related plant parameters significantly. Water content, dry mass, photosynthetic pigments, certain osmolytes, and enzymatic antioxidants increased significantly, according to our findings. The use of sewage sludge as an organic source resulted in a larger increase in the Zn, Cd, Cu and Ni content of the plants studied. Heavy metal buildup is reduced in colonization plants with endophytic fungus. We recommended the use of endophyte *T. pinophilus* for phytoremediation of sewage sludge which can be used as a bio-fertilizer because it had a positive effect on plant growth, related plant parameters, soil biochemical and physical properties. It was an effective technique for sustainable agriculture in heavy metal-contaminated lands. More studies should be done in phytoremediation with different plants.

## Figures and Tables

**Table 1:** Treatments used in the cultivation of *T. aestivum*.

No	Treatments	Treatments Abbreviations
1	Sandy soil without sewage sludge and without endophytic fungus (100% sandy soil)	T1
2	75% soil + 25% sewage sludge	T2
3	50% soil +50% sewage sludge	T3
4	25% soil + 75% sewage sludge	T4
5	100% sandy soil + endophytic fungus as mentioned in Fungal Inoculum Preparation	T5
6	75% soil + 25% sewage sludge + endophytic fungus	T6
7	50% soil + 50% sewage sludge + endophytic fungus	T7
8	25% soil + 75% sewage sludge + endophytic fungus	T8

**Table 2:** GenBank nucleotide accessions of *Talaromyces pinophilus* strains from the GenBank based on ITS sequences used for phylogenetic analysis.

No	Morphospecies	Strian	Sequence (bp)	Host	Location	GenBank Accessions
1	*Talaromyces pinophilus*	SVU3:83523	574	*Rosmarinus officinalis*	Egypt	MW695526
2	*Talaromyces pinophilus*	Y. H. Yeh I0609	595	*Ipomoea*	Taiwan	MK336445
3	*Penicillium pinophilus*	FKI-3864	596	-	Japan	AB455516
4	*Talaromyces pinophilus*	17F4103	600	-	Japan	MT093464
5	*Talaromyces pinophilus*	Y. H. Yeh I0520	584	*Ipomoea*	Taiwan	MK336630
6	*Talaromyces pinophilus*	C.W. Hsieh CHD152	591	*Platostomapalustre*	Taiwan	MH777072
7	*Talaromyces pinophilus*	1–95	6,009,755	-	China	CP017345

**Table 3:** Gibberellic acid and abscisic acid content of *T. aestivum* plants grown on mixtures of sandy soil and sewage sludge and the same treatment mixed with *T. pinophilus* (100% sandy soil (T1), 75% sandy soil + 25% sewage sludge (T2), 50% sandy soil + 50% sewage sludge (T3), 25% sandy soil + 75% sewage sludge (T4), 100% sandy soil + *T. pinophilus* (T5), 75% sandy soil + 25% sewage sludge + *T. pinophilus* (T6), 50% sandy soil + 50% sewage sludge + *T. pinophilus* (T7), 25% sandy soil + 75% sewage sludge + *T. pinophilus* (T8).

Treatment	Gibberellic Acid (μg mL^−1^)	Abscisic Acid (μg mL^−1^)
T1	4.11	3.05
T2	0.41	0.09
T3	2.11	2.19
T4	0.00	0.00
T5	2.00	5.14
T6	5.36	0.00
T7	2.78	0.00
T8	5.22	7.13

**Table 4:** Soil pH, organic matter (OM) and cadmium (Cd), copper (Cu), zinc (Zn) and nickel (Ni) contents for different mixtures of sandy soil and sewage sludge. 100% sandy soil (T1), 75% sandy soil + 25% sewage sludge (T2), 50% sandy soil + 50% sewage sludge (T3), 25% sandy soil + 75% sewage sludge (T4), 100% sandy soil + *T. pinophilus* (T5), 75% sandy soil + 25% sewage sludge + *T. pinophilus* (T6), 50% sandy soil + 50% sewage sludge + *T. pinophilus* (T7), 25% sandy soil + 75% sewage sludge + *T. pinophilus* (T8). Different letters indicate significant differences between treatments before planting or after harvesting (ANOVA, *p* < 0.05; Duncan test, *p* < 0.05) and asterisks indicate significant differences between before planting and after harvesting (*t*-test, *p* < 0.05).

Treatments		T1	T2	T3	T4	T5	T6	T7	T8
pH	Before	8.15 ^f^^,^*	7.82 ^e^	7.57 ^d^ *	7.56 ^d,^*	7.28 ^c^	6.86 ^b^	6.92 ^b^	6.46 ^a^
	After	7.97 ^d^	7.63 ^c^	7.08 ^b^	6.91 ^ab^	6.72 ^a^	6.75 ^ab^	6.93 ^ab^	6.69 ^a^
OM (%)	Before	2.73 ^a,^*	3.60 ^a^ *	6.53 ^b,^*	8.44 ^c,^*	3.76 ^a,^*	5.74 ^b,^*	16.16 ^d,^*	22.52 ^e,^*
	After	2.07 ^a^	2.87 ^b^	4.97 ^d^	6.18 ^f^	2.68 ^ab^	3.85 ^c^	12.72 ^g^	17.23 ^h^
Na (mg g^−1^)	Before	1.73 ^a^	2.08 ^a^	3.54 ^b^	4.94 ^c^	1.05 ^a^	3.32 ^b,^*	9.58 ^e,^*	6.37 ^d,^*
	After	1.16 ^a^	2.16 ^b^	3.07 ^c^	4.34 ^d^	0.84 ^a^	3.41 ^c^	6.65 ^e^	8.99 ^f^
K (mg g^−1^)	Before	3.63 ^a^	6.04 ^b,^*	7.99 ^c,^*	9.52 ^d,^*	6.09 ^b,^*	10.14 ^d^	15.27 ^e^	25.29 ^f,^*
	After	2.92 ^a^	4.85 ^b^	6.26 ^c^	7.13 ^cd^	3.59 ^ab^	8.28 ^d^	15.00 ^e^	19.23 ^f^
Ca (mg g^−1^)	Before	7.12 ^a,^*	10.39 ^b,^*	14.38 ^c^	16.95 ^d,^*	13.10 ^c,^*	18.29 ^d,^*	20.80 ^e,*^	28.86 ^f,^*
	After	4.96 ^a^	8.31 ^b^	10.63 ^d^	12.95 ^e^	9.65 ^c^	11.01 ^d^	16.01 ^f^	19.73 ^g^
Mg (mg g^−1^)	Before	3.05 ^a,^*	4.09 ^b^	7.04 ^d^	8.74 ^e,^*	5.88 ^c^	9.75 ^f^	15.79 ^g,^*	19.24 ^h,^*
	After	1.50 ^a^	3.20 ^ab^	4.74 ^b^	6.95 ^c^	4.20 ^b^	7.37 ^c^	10.13 ^d^	13.93 ^e^
Cd (µg g^−1^)	Before	0.30 ^b^	0.58 ^c^	0.79 ^e^	1.00 ^f^	0.22 ^a,^*	0.32 ^b,^*	0.55 ^c,^*	0.70 ^d,^*
	After	0.23 ^a^	0.53 ^b^	0.71 ^c^	0.93 ^d^	0.19 ^a^	0.24 ^a^	0.48 ^b^	0.55 ^b^
Cu (µg g^−1^)	Before	10.44 ^d^^,^*	12.49 ^e,^*	18.94 ^f^^,^*	20.94 ^g^^,^*	4.90 ^a^^,^*	5.86 ^ab^^,^*	6.69 ^b^^,^*	9.05 ^c^^,^*
	After	7.34 ^d^	10.27 ^e^	14.37 ^f^	15.18 ^f^	2.25 ^a^	3.57^b^	5.05 ^c^	6.79 ^d^
Zn (µg g^−1^)	Before	40.91 ^d^^,^*	45.44 ^f^^,^*	55.50 ^g^^,^*	77.21 ^h^^,^*	22.01 ^b^^,^*	20.19 ^a^^,^*	32.63 ^c^^,^*	43.92 ^e^^,^*
	After	31.79 ^c^	29.78 ^c^	49.63 ^e^	70.49 ^f^	16.32 ^a^	14.17 ^a^	23.06^b^	34.03^d^
Ni (µg g^−1^)	Before	26.85 ^d^^,^*	32.31 ^e^^,^*	43.23 ^f^^,^*	61.95 ^g^^,^*	13.54 ^a^^,^*	16.71 ^b^^,^*	20.72 ^c^^,^*	25.84 ^d^^,^*
	After	21.78 ^d^	27.72 ^e^	36.12 ^f^	50.27 ^g^	10.78 ^a^	13.48 ^b^	16.62 ^c^	20.38 ^d^

**Table 5:** Bioconcentration factor (BCF) and translocation factor (TF) of metals under different soil andsewage sludge mixtures: 100% sandy soil (T1), 75% sandy soil + 25% sewage sludge (T2), 50% sandy soil + 50% sewage sludge (T3), 25% sandy soil + 75% sewage sludge (T4), 100% sandy soil + *T. pinophilus* (T5), 75% sandy soil + 25% sewage sludge + *T. pinophilus* (T6), 50% sandy soil + 50% sewage sludge + i (T7), 25% sandy soil + 75% sewage sludge + *T. pinophilus* (T8).

	Zn	Cd	Ni	Cu
Bioconcentration factor (BCF)				
T1	0.958	0.689	0.363	0.435
T2	1.410	0.790	0.424	0.633
T3	1.289	0.832	0.391	0.497
T4	1.438	0.804	0.334	0.527
T5	2.111	0.769	0.690	0.869
T6	2.613	1.116	0.714	0.889
T7	1.892	0.813	0.641	0.939
T8	1.861	0.670	0.489	0.531
Translocation factor (TF)				
T1	0.264	0.752	1.022	0.536
T2	0.613	0.768	0.660	0.886
T3	0.683	0.720	0.832	1.081
T4	0.755	0.953	0.722	0.528
T5	0.597	0.895	1.015	0.464
T6	0.729	0.501	0.643	0.708
T7	0.524	0.894	0.729	0.918

## Data Availability

The datasets generated and/or analyzed during the current study are available from the corresponding author upon reasonable request.
